# Local Anæsthesia

**Published:** 1878-10

**Authors:** 


### Editorial.

### LOCAL ANÆSTHESIA.

I have for about two years been using a preparation for local anæsthesia, devised by Dr. C. Von Bonhorst.

In many cases of extraction of teeth, and for other operations in the mouth—cutting, lancing abscesses, etc.—it has been very valuable indeed. In some cases it was not effective—giving little or no relief.

The Doctor has recently made an improvement in the composition that renders it more prompt in action, and effective in a much larger number of cases, and so more valuable. One familiar with its use and efficiency would be unwilling to be without it.

There may be other preparations just as good, bnt I know of none as effective and so little objectionable in taste and odor.

It is worthy of a trial by every dentist.

